# Enrichment and expansion with nanoscale artificial antigen presenting cells for T cell adoptive immunotherapy

**DOI:** 10.1186/2051-1426-2-S3-P34

**Published:** 2014-11-06

**Authors:** Karlo Perica, Joan Bieler, Christian Schuetz, Juan Varela, Mathias Oelke, Jonathan Schneck

**Affiliations:** 1 Johns Hopkins School of Medicine, Baltimore, Maryland, United States; 2 Johns Hopkins School of Medicine, Department of Pathology, Institute for Cell Engineering, Baltimore, Maryland, United States

## 

Adoptive T cell therapy can mediate durable regression of cancer [1]. While pre-existing anti-tumor responses can only be cultured from a minority of cancer patients [2], T cells specific for a wide variety of tumor antigens can be generated by stimulation of naive precursor cells with tumor antigen [3]. This culture process relies on autologous antigen presenting cells and feeder cells, which are complex biologics that must be generated for each individual patient [4], significantly increasing the cost and complexity of adoptive immunotherapy.

To quickly generate large numbers of functional tumor-specific T cells from naïve T cell precursors, we developed a T cell Enrichment+Expansion strategy using paramagnetic, nanoscale artificial Antigen Presenting Cells (nano-aAPC), which are capable of enriching rare tumor-specific T cells in a magnetic column and activating them. We generated up to 150,000 total Trp2-specific cells in only one week from 10 million polyclonal CD8 lymphocytes containing approximately 10 precursor cells [5]. Similar results were obtained for other tumor and model antigens, including the human tumor antigens A2-NY-ESO1 and A2-MART1. We further demonstrate that removing irrelevant bystander cells by enrichment confers a significant survival and proliferation advantage to tumor-specific T cells both during *in vitro *culture and after adoptive transfer *in vivo*. Streamlining the generation of large numbers of high-frequency tumor-specific T cells in a cost effective, reproducible fashion through Enrichment+Expansion could be a powerful addition to autologous tumor immunotherapy protocols.
